# Editorial: Emerging perspectives on probiotics, prebiotics, and synbiotics for prevention and management of chronic disease

**DOI:** 10.3389/fnut.2023.1309140

**Published:** 2023-10-26

**Authors:** Arun K B, P. Nisha, Alberto Finamore

**Affiliations:** ^1^Department of Life Sciences, CHRIST (Deemed to be University), Bangalore, Karnataka, India; ^2^CSIR - National Institute for Interdisciplinary Science and Technology, Thiruvananthapuram, Kerala, India; ^3^Academy of Scientific and Innovative Research (AcSIR), Ghaziabad, India; ^4^Council for Agricultural Research and Economics—Research Centre for Food and Nutrition (CREA—Food and Nutrition), Rome, Italy

**Keywords:** prebiotics, probiotics, synbiotics, chronic diseases, prevention and management

The human gastrointestinal system is a complex ecosystem housing approximately 10^14^ microorganisms crucial for gut health and immune function (1). These microorganisms produce metabolites able to influence several body processes in health and disease. An imbalance in this microbial community, termed dysbiosis, it has been shown to be associated to various health issues, including gastric cancer, diabetes, liver disorders, cardiovascular diseases, obesity, inflammatory bowel disease, and colorectal cancer. Several studies have explored probiotics, prebiotics, and synbiotics as potential remedies to restore a healthy gut microbiome and combat dysbiosis. The goal of this topic is to bring together new research articles that illuminate the evolving perspectives on these bioactive components for the prevention and management of chronic diseases. In this research area, a Research Topic of 8 studies has been published. These studies are making substantial contributions to the continuously evolving field of research focused on exploring the potential advantages of probiotics and prebiotics and their impact on human health.

Cardiovascular diseases (CVDs) remain a major global health concern, with hypercholesterolemia being a prominent risk factor. Traditional therapeutic approaches often involve chemotherapeutic agents with several side effects. The article by Aswal et al. explores the genome of *Enterococcus faecium* LR13, a cholesterol-assimilating probiotic strain, and compares it with other *E. faecium* strains. The study identifies a unique protein in LR13 that may contribute to its hypocholesterolemic effects. These findings hold promise for the development of novel biotherapeutics against CVDs, emphasizing the potential role and mechanisms of action of probiotics in managing chronic diseases (Aswal et al.).

In the study by Wei et al., the effects of postbiotics on constipation have been evaluated. Constipation is a prevalent gastrointestinal disorder, especially among the elderly. Wei et al. investigated the effects of a postbiotic derived from hawthorn-probiotic in a model of aged mice with constipation, they observed that postbiotics were able to improve intestinal function and modulate gut microbiota showing promising effect of postbiotics in alleviating constipation. This research highlights the potential effects of postbiotics in addressing gastrointestinal issues associated with aging, offering a novel approach to managing chronic digestive problems (Wei et al.). Constipation is a common childhood issue and several studies have evaluated the effects of probiotics to alleviate constipation, in this regard, Dong et al. reviewed various systematic reviews and meta-analyses to examine the collective evidence regarding the effects of probiotics on constipation. The analysis indicates that probiotic intake in children significantly improves treatment success rates and reduces the recurrence of constipation. This underscores the role of probiotics as a safe and effective approach to managing chronic constipation in children (Dong et al.).

Tailor et al. have given a systematic review exploring the impact of dietary fiber on periodontal disease in animal models. Periodontal disease, a chronic inflammatory condition affecting oral health, has been linked to dietary factors, including fiber intake. The review summarized that increased dietary fiber intake may reduce inflammation and alveolar bone loss in periodontitis. While these findings are preliminary, they underline the potential of dietary interventions involving prebiotics in managing chronic oral health issues (Tailor et al.).

Coeliac disease is an autoimmune condition triggered by gluten consumption. Jenickova et al. have studied the effect of specific probiotic strains (*Lactiplantibacillus plantarum* and *Lacticaseibacillus paracasei*) supplementation on the fecal metabolome in children with coeliac disease. They showed significant changes in the metabolome after probiotic consumption, emphasizing that probiotics may influence metabolic processes in coeliac disease. This research offers valuable insights into the role of probiotics in managing chronic autoimmune disorders (Jenickova et al.).

Plants have long been regarded as dependable sources of therapeutic agents for various illnesses, and a significant number of synthetic drugs find their origins, whether directly or indirectly, in the plant kingdom. Diabetes mellitus type II is a chronic condition characterized by high blood sugar levels. Findings from recent research highlight the potential of plants and their products to exhibit notable effectiveness in managing diabetes (2). Regarding the last matter, in this Research Topic, a randomized controlled pilot study that explores the potential hypoglycemic effect of plants in humans with diabetes has been submitted. The authors performed a randomized controlled pilot study to evaluate the hypoglycemic effect of Kombucha tea in humans affected by diabetes. Kombucha tea is a beverage obtained from the fermentation of sweetened tea with a synbiotic mixture and it results in reach in lactic acid and acetic acid bacteria with probiotic activity. Despite the limitation of the sample size, the results indicate that kombucha beverage consumption is associated with reduced fasting blood glucose levels, suggesting that kombucha could be a promising dietary addition for individuals with diabetes, highlighting the potential of probiotics in managing chronic metabolic disorders (Mendelson et al.).

Environmental enteropathy is a chronic intestinal condition linked to poor sanitation and chronic exposure to fecal contamination. This study explores the potential effect of fermented food-derived *Limosilactobacillus fermentum* strains isolated from fermented rice water to combat environmental enteropathy in an *in vitro* and *in vivo* model. *L. fermentum* showed demonstrated probiotic properties, antagonistic effects against pathogens, and immunomodulatory effects. These findings offer hope for addressing chronic intestinal disorders prevalent in low-income countries through targeted nutritional interventions (Prakash et al.). Colorectal tumorigenesis is associated with imbalances in gut microbiota and different signaling pathways. The research by Lin et al. investigates the role of *Turicibacter* fermentation and *Antrodia camphorata* supplementation in inhibiting tumorigenic pathways and promoting apoptosis in colorectal cells by affecting *Wnt* signaling and promoting ROS-mediated apoptosis in an *in vitro* model. The results suggest that *Turicibacter* fermentation enhances the anti-cancer effects of *A. camphorata* supplementation, highlighting the potential of synbiotic approaches in managing chronic diseases like colorectal cancer (Lin et al.).

The articles presented in this editorial collectively emphasize the emerging perspectives on probiotics, prebiotics, and synbiotics in the prevention and management of chronic diseases. These bioactive components offer promising avenues for addressing a wide range of health conditions, from cardiovascular diseases and diabetes to periodontal disease, coeliac disease, and constipation. As research in this field continues to expand, it is evident that the microbiome plays a critical role in the pathogenesis and management of chronic diseases, offering innovative approaches to enhance human health and wellbeing.

## Author contributions

AKB: Conceptualization, Writing—original draft. PN: Writing—review & editing. AF: Writing—review & editing.
